# Immune radiobiology

**DOI:** 10.1186/s12967-021-02928-w

**Published:** 2021-06-10

**Authors:** Claire Vanpouille-Box

**Affiliations:** 1 Department of Radiation Oncology, Stich Radiation Oncology, Weill Cornell Medical College, 525 East 68th Street, Box #169, New York, NY 10065 USA; 2 Sandra and Edward Meyer Cancer Center, New York, NY 10065 USA

## Main text

We are thrilled to announce the launch of a new section in the *Journal of Translational Medicine* entitled “*Immune RadioBiology*”. This section is interested in innovative research that investigate the crosstalk between ionizing radiations (IR), the tumor microenvironment (TME) and host immunity. Studies considered for publication include (but not limited to) those investigating the mechanisms of radiation-induced anti-tumor immune responses, the immune-stimulatory and immuno-suppressive properties of ionizing radiation from all sources (X-rays, FLASH-RT, protons, carbon ion and radionuclides), biomarkers predictive of immune response, immune escape mechanisms elicited by IR and radioresistance. Importantly, we are welcoming translational and clinical studies assessing the combination of IR with immunotherapies (IT) with an emphasis on clinical response and outcome.

The ability of IR to modulate host immune responses has been described forty years ago with seminal work from Stone who demonstrated that the efficacy of radiation therapy was dependent on the presence of T cells [1]. While these findings have been overlooked for a long-time, the advent of modern IT together with the growing evidence of the immunoadjuvant properties of IR has brought radiation therapy to the limelight [2, 3].

The main concept is to exploit the immunogenic potential of IR to jumpstart host immune responses with the ultimate goal to raise the tail of cancer patient survival curves treated with immunotherapies [2, 4]. The rationale for the use of IR as an immune adjuvant originates from extensive preclinical studies demonstrating that IR elicit an immunogenic cell death (ICD) and a type I interferon (IFN-I) response to generate antigen specific T cell killing [2]. These two non-mutually exclusive IR-induced biologic processes rely on (1) the coordinate release of damaged associated molecular pattern (DAMPs) molecules subsequent to oxidative endoplasmic reticulum (ER) stress; namely the surface exposure of calreticulin, the secretion of ATP and the release of high mobility group box 1 (HMGB1) [5–7] and (2) the activation of nucleic acid sensors pathways such as the CGAS/STING (cyclic GMP-AMP synthase/stimulator of interferon genes) and/or the retinoic acid inducible gene-I (RIG-I)-like receptors (RLRs) [8–10]. Importantly, not only IR increases adjuvanticity of an irradiated tumor, but it can also promote tumor control by exposing immunogenic mutations to the immune system [11, 12], which further support systemic immunity.

Despite these immuno-stimulatory properties, IR not always convey immunogenicity. Reasons for such occurrence rely on the concomitant induction of immune resistance mechanisms of the TME, including (but not limited to) the induction of hypoxia, the activation of immunosuppressive cytokines such as transforming growth factor-beta (TGFβ) [13] or the recruitment of regulatory T cells [14, 15]. Consequently, while IR has the potential to become a game changer in the field of immuno-oncology due to its capability to shape immune responses, mechanistic insight from preclinical models underscore the intricacy of host and tumor responses. Notably, immunogenicity of IR is likely dependent on tumor intrinsic factors, the radiosensitivity of the tumor, the composition of the TME and parameters pertaining to IR itself (i.e. radiation dose, dose rate, fractionation and source of IR). Indeed, the optimal IR dose and regimen to initiate anti-tumor immunity is currently debated with conflicting results that has been reported. Notably, a single dose of 20 Gy was unable to jumpstart anti-tumor immunity in a murine breast cancer model [10], while it was reported as a potent immune adjuvant when combined with immune checkpoint blockers (ICB) in a preclinical model of melanoma [16]. These opposed results obtained in two distinct mice models, suggest that factors from the TME may play a role in influencing the immunogenicity of IR. Supporting this notion, a recent study from Sia demonstrated that radiation dose per fraction (DPF) rather than the biological effective dose (BED) determined the ability of RT to synergize with ICB and initiate local anti-tumor CD8 + T cells, an effect that was influenced by the induction of Tregs [15].

Aside RT dose and fractionation, another parameter that has been largely ignored is the type of IR that can be utilized to trigger anti-tumor immunity. High linear energy transfer (LET) radiations, (alpha- and beta- radionuclides, protons, carbon ion) deposit a large amount of energy over a short distance therefore causing greater DNA damages as compared to low LET radiation (e.g. γ-irradiations and X-rays). Although immune-stimulatory properties of high LET IR needs to be defined, preliminary data indicate that alpha-RT (e.g. bismuth-213) and beta-IR (e.g. rhenium-188) act as an ICD inducer capable to stimulate adaptive immunity in experimental models of colorectal cancer and glioblastoma [17, 18]. Along similar line, seminal work assessing the immunogenic properties of the ultra-high dose-rate radiotherapy (FLASH-RT; > 40 Gy/s) suggest that FLASH-RT hold great promises in stimulating anti-tumor immunity while preserving excessive irradiation to healthy tissues in a murine glioblastoma model [19].

Overall, extensive work over the last two decades revealed that the immunoadjuvant potential of IR is heavily influenced by (1) tumor intrinsic factors, (2) the composition of the TME, (3) the induction of immunosuppressive signals post IR, (4) dose per fraction, (5) sequencing between IR and IT, and (6) the source of IR. Consequently, translation of these findings to the clinic has been more complicated than predicted which call for longitudinal immunophenotyping and multi-factorial bioinformatic analysis to determine the kinetic of immune response in IR-IT combinatorial approaches.

By providing a platform that merges expertise of radiobiology and immunology for the communication and dissemination of innovative and high-standard peer-review studies, this new section of *Journal of Translational Medicine* will reflect the fast-evolving field of immune radiobiology. The editorial board is eager to receiving your contributions.
